# The Effects of Remote Activity Monitoring on Family Caregivers of People Living With Dementia Over an 18-Month Period

**DOI:** 10.1093/geroni/igab046.009

**Published:** 2021-12-17

**Authors:** Joseph Gaugler, Rachel Zmora, Lauren Mitchell, Jessica Finlay, Christina Rosebush, Colleen Peterson, Manka Nkimbeng, Zachary Baker

**Affiliations:** 1 University of Minnesota, Minneapolis, Minnesota, United States; 2 Emmanuel College, Boston, Massachusetts, United States; 3 University of Michigan, Ann Arbor, Michigan, United States

## Abstract

Technology interventions for older persons and long-term care are generally utilized as real-time data capture tools to complement clinical or family care for older persons or as interventions themselves designed to improve important dementia care outcomes. Although research on novel technological interventions for people with Alzheimer's disease and related dementias (ADRD) and their family caregivers has grown considerably in the past two decades, much of this work continues to focus on design, feasibility, and acceptability (with a need for conceptual refinement in these areas) and less on controlled outcome studies. The objective of this experimental mixed methods demonstration was to determine the 18-month effectiveness of remote activity monitoring (RAM) technology in improving outcomes among family caregivers of community-dwelling persons with dementia. We used an embedded experimental mixed methods design, collecting qualitative data within the structure of a traditional randomized controlled trial ([QUAN+qual]→QUAN) over an 18-month period for 171 dementia caregivers. Change in caregiver self-efficacy, sense of competence, and caregiver distress served as the main quantitative outcomes of interest. Individual growth curve models indicated that the RAM technology did not have direct effects on caregiving outcomes, and although the qualitative findings indicated several potential moderators of RAM effectiveness on caregiving outcomes, the inclusion of these qualitatively-identified moderators did not result in statistically significant (p < .05) effects. Ensuring effective human care management alongside RAM technology may help to overcome the barriers reported by dementia caregivers in this demonstration study.

